# Updated clinical overview on cardiac laminopathies: an electrical and mechanical disease

**DOI:** 10.1080/19491034.2018.1489195

**Published:** 2018-10-03

**Authors:** G. Peretto, S. Sala, S. Benedetti, C. Di Resta, L. Gigli, M. Ferrari, P. Della Bella

**Affiliations:** a Department of Cardiac Electrophysyology and Arrhythmology, IRCCS San Raffaele Hospital and University, Milan, Italy; b Laboratory of Clinical Molecular Biology and Cytogenetics, IRCCS San Raffaele Hospital and University, Milan, Italy; c Genomic Unit for the diagnosis of human pathologies, Division of Genetics and Cellular Biology, IRCCS San Raffaele Hospital and University, Milan, Italy

**Keywords:** LMNA, arrhythmias, cardiomyopathy, heart failure, sudden cardiac death, lamin, genetics

## Abstract

Cardiac laminopathies, associated with mutations in the LMNA gene, encompass a wide spectrum of clinical manifestations, involving electrical and mechanical alterations of cardiomyocytes. Thus, dilated cardiomyopathy, bradyarrhythmias and atrial or ventricular tachyarrhythmias may occur in a number of combined phenotypes. Nowadays, some attempt has been made to identify clinical predictors for the most life-threatening complications of LMNA-associated heart disease, i.e. sudden cardiac death and end-stage heart failure. The goal of this manuscript is to combine the most recent evidences in an updated review to show the state-of-the-art of such a complex disease group. This is supposed to be the starting point to collect more data and design new *ad hoc* studies to identify clinically useful predictors to stratify risk in mutation carriers, including probands and their asymptomatic relatives.

## Introduction

The lamin A/C gene (LMNA), located in human chromosome 1q22, codes by alternative splicing for lamin A and C, proteins of the nuclear lamina playing a key role in nuclear architecture maintenance, chromatin organization and gene expression regulation [1,2]. Lamin A and C are predominantly expressed in terminally differentiated cells, including cardiomyocytes [3]. Thus, several mutations in the LMNA gene have been associated with a cardiac phenotype, characterized by a very peculiar co-existence of structural abnormalities and electrical instability. As a consequence, progressive dilated cardiomyopathy (DCM), atrioventricular conduction disorders and both atrial and ventricular tachyarrhythmias are commonly found in these patients [4], leading to severe heart failure (HF) or sudden cardiac death (SCD) as extreme manifestations, occurring usually by middle age [5]. Of note, electrical dysfunction is reported to precede mechanical disease, although the delay interval ranges from a few years to more than a decade according to different published experiences [6].

From a pathophysiological viewpoint, myocardial fibrosis is thought to be responsible for the development of both electrical instability and mechanical impairment [7]. Some consideration about fibrosis should be taken into account. First, it is probably a primary phenomenon, due to mutated gene expression; in fact, not differently from other genetically-determined cardiomyopathies, it initially involves interstitium; moreover, differently from seconday fibrosis, it is apparently unrelated to the renin-angiotensin-aldosterone axis activation, since it has been shown that plasma renin activity and aldosterone levels are not signiﬁcantly increased in LMNA mutation carriers [8]. Second, it may explain both conduction system disease and tachyarrhythmias tipically associated to LMNA-associated DCM; in fact, previous autopsy studies have shown the location of fibrosis within the interventricular septum, near the region of the conduction system [9]; accordingly, more recent works have found the majority of the clinically-relevant ventricular tachycardias originate from the septum itself [10]. Third, although inconstantly, fibrosis may involve skeletal muscle too; in fact, LMNA-related autosomal dominant type 2 Emery-Dreifuss muscular dystrophy (EDMD) and type 1B limb-girdle muscular dystrophy (LGMD) show often cardiac involvement with both structural heart disease and arrhythmias [11].

**Figure 1.**
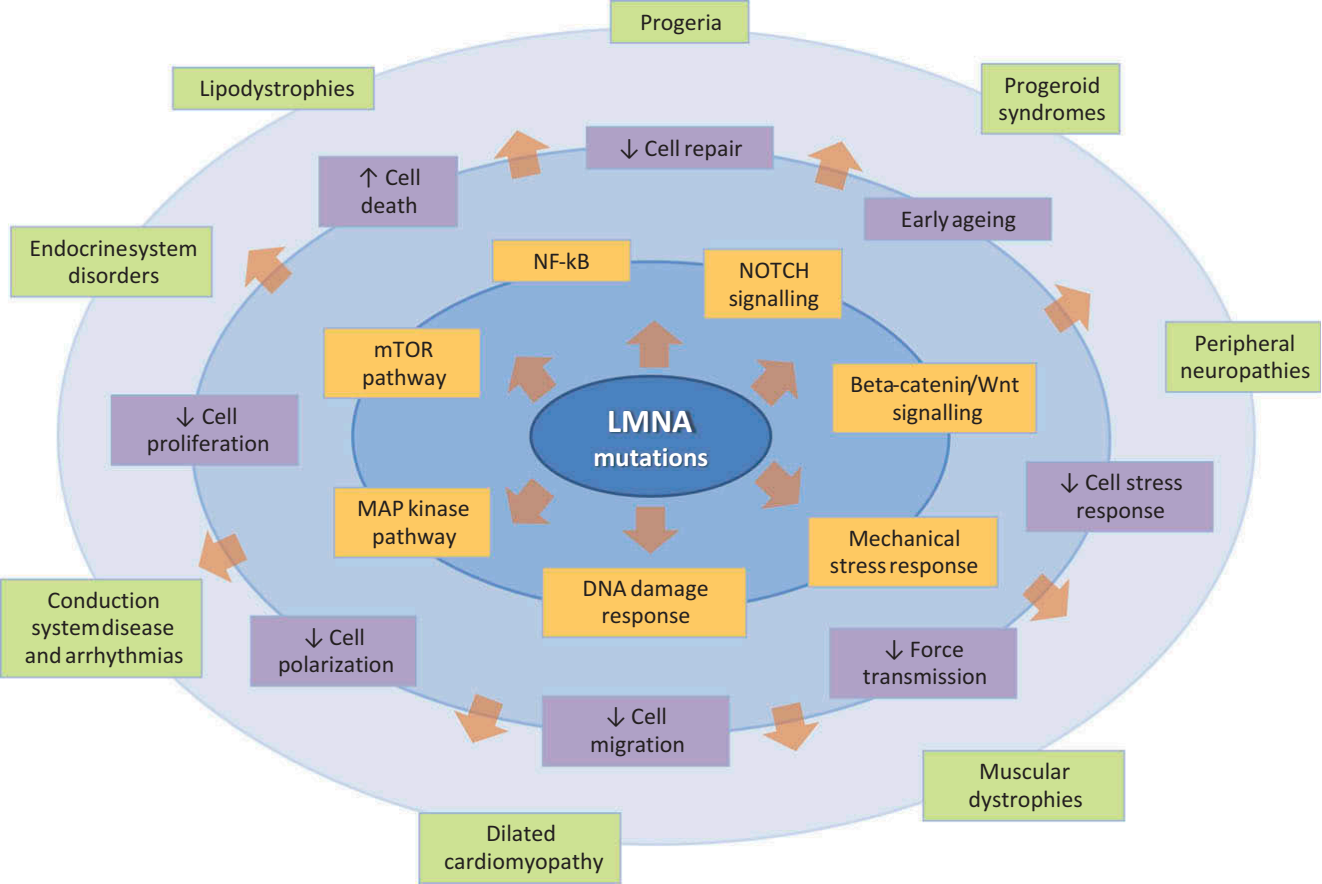
Pathophysiological overview of human laminopathies. The figure shows the complexity of pathophysiological mechanisms that, from mutations in the LMNA gene, finally lead to a broad spectrum of possible clinical phenotypes, including but not limited to heart disease. In particular, genotype-to-phenotype transition is divided into three levels represented by concentric lines: the internal one (molecular level) represents molecular pathways more commonly involved in LMNA gene mutations; the intermediate one (cellular level) summarizes the subsequent alterations of cell functions; the external one (phenotypic level) shows the main clinical syndromes associated to LMNA gene mutations, with a wide spectrum of possible overlapping phenotypes.

More in general, since pathogenic LMNA gene variants have also been identified in several disorders of striated muscle, nerve, adipose, and vascular tissue, collectively referred to as laminopathies (Figure 1) [3], heterogeneous and multisystemic clinical involvement may appear, generating a number of so-called overlapping syndromes [12].

## Structural heart disease

DCM is a major cause of HF and cardiac death worldwide, with an estimated prevalence or 1:500 or greater [13]. Although only 0.5–5% of patients with DCM show LMNA pathogenic variants by genetic analysis, mutations in lamin A/C are reported in up to 10% of the familial cases, and up to 33% of the cases of DCM with atrioventricular conduction disorders [6,14]. The pattern of inheritance of LMNA-related DCM is autosomal dominant, so that heterozygotes are often affected, even if with variable expressivity [15]. Age-related penetrance has been reported, with cardiac phenotype starting between third and fourth decades and reaching 90%-95% by the seventh decade [6]. Furthermore, the manifestation of cardiac disease might be different between men and women [16]. Of note, differently from other idiopathic DCMs, those associated with LMNA mutations are characterized by a relatively greater occurrence of arrhythmias, including conduction system defects and a wide range of tachyarrhythmias [4]. Occasionally, individuals with LMNA-related DCM also manifest signs or symptoms of skeletal myopathy, presenting with either nonspecific muscle weakness, definite neuromuscular syndromes (basically type 2 EDMD, type 1B LGMD, or LMNA-related congenital muscular dystrophy, with a considerable clinical overlap) or isolated creatine kinase elevation (usually non-superior to 5-times upper normal value) [11]. Of note, cardiac phenotype usually follows, but in some cases may precede skeletal myopathy or show an independent natural course [17]. However, early diagnosis of DCM is often difficult, because the left ventricle (LV) dilatation is typically mild at the beginning, and may remain asymptomatic in most of the patients for many years. By converse, an early cardiomyopathy, characterized by an even severe decrease in LV ejection fraction (LVEF) in spite of normal or near-normal LV volumes has been also described [18]. Finally, isolated or combined right ventricular dilatation, dysfunction and even fibrofatty replacement have been reported, so that a phenotype overlapping with arrhythmogenic right ventricular cardiomyopathy is a further possible manifestation [19,20]. Similarly to other primary cardiomyopathies, it has been also shown that a previous history of competitive sport can produce a deleterious effect also in LMNA-related DCM [21].

Primary myopathy may involve the atria too, leading to chamber dilatation, mechanical dysfunction, electrical conduction delay and atrial fibrillation (AF). Although rarely, patients may also develop an atrial paralysis, a severe modality of cardiac compromise typical for cardiac laminopathies [22]. Whichever the onset, LMNA-related DCM has a more aggressive clinical course as compared with other forms of familial DCMs, due to the high rate of end-stage HF leading to heart transplantation (HTx) [18], conduction disease and death [4]. Recent report suggest that, in the presence of even mild systolic dysfunction (LVEF below 50%), the progression to end-stage HF adds up to 57% at 7 years [23]. Consistently, up to 9% of the patients undergoing HTx for DCM show LMNA mutations [24]. Of note, even among patients without LV dysfunction or HF at presentation, almost ¼ develop new onset HF or LV dysfunction, and 7% reaches end-stage HF at 7 years of follow-up [23].

## Electrical heart disease

The earliest finding in LMNA-related heart disease is usually a conduction system defect, alone or associated with atrial or ventricular tachyarrhythmias [25]. In its typical form, electrical disease appears with mild arrhythmias before or during the third decade of life [26]. After the age of 30, it is reported that up to 92% of patients bearing LMNA gene mutations present arrhythmias, including non-threatening manifestations like 1^st^ degree AVB, frequent premature ventricular contractions (PVCs) or non-sustained ventricular tachycardia (NSVT) [26]. These disorders are due either to impulse formation, or more often to impulse conduction [27]. In particular, myocardial ﬁbrosis has been identiﬁed in the heart of LMNA mutation carriers experiencing arrhythmias or conduction disturbances, irrespective of LV dilation and/or dysfunction [7].

**Conduction system** involvement usually starts with disease of the sinus node and/or atrioventricular node, that can manifest as sinus bradycardia, sinus sick syndrome with node arrest and junctional escape rhythms, or any-degree atrioventricular block (AVB) [6]. During the earliest phase, it is common to observe mild conduction system disorders like first degree AVB or inter/intra-ventricular conduction delays including complete and incomplete bundle branch blocks with or without signs of structural heart disease [28]. By ageing, second or third degree AVBs typically occur, possibly leading to SCD [29]. Overall, it is estimated that 44% of patients will eventually need pacemaker implantation after the age of 30 because of bradyarrhythmias [26]. Nevertheless, these patients are not protected from the tachyarrhythmic causes of SCD, as up to 50% of electrical sudden deaths happen in patients who already have a pacemaker [22].

**Supraventricular tachyarrhythmias** including AF, atrial flutter and focal atrial tachycardia occur as expression of atrial disease. In particular, AF has been shown to progresses from paroxysmal to persistent or permanent forms (45%), and to be associated with high incidence of thromboembolic events (10% at 7 years) [23].

**Ventricular arrhythmias** (VA), including frequent PVCs, ventricular tachycardia (VT) and ventricular fibrillation (VF), are reported as typical manifestations of LMNA-related heart disease [18,22]. Among them, malignant VAs like sustained VT and VF are actually the leading cause of SCD in LMNA mutation carriers with cardiac involvement [2]. Unfrequently, however, life-threatening arrhythmias appear as the ﬁrst clinical manifestation, since non-severe arrhythmias or at least mild structural heart disease usually precedes their occurrence [30]. However, both incidence and prognostic impact of SCD are more common than end-stage mechanical dysfunction: in fact, SCD in cardiac laminopathies is at least four times more frequent than death due to HF and in 50% of cases it happens before the stage of symptomatic structural heart disease [4]. Subsequently, the only reliable treatment for the prevention of SCD is the implantable cardioverter-defibrillator (ICD), as it will be discussed below [26].

## Diagnosis

**Clinically**, LMNA-related cardiomyopathy should be suspected in individuals with a clinical diagnosis of familial idiopathic DCM, especially in association with conduction system disease and/or supraventricular or ventricular arrhythmias It is worth noting, however, that isolated electrical disease may precede by several years signs and symptoms of mechanical disease [26]. Moreover, since DCM may also be present in asymptomatic individuals, diagnosis may be discovered during a routine medical evaluation conducted for another reason or by clinical screening of at-risk relatives. Key alarm **symptoms** should always be investigated, addressing both mechanical disease (shortness of breath, dyspnea on exertion, paroxysmal nocturnal dyspnea) and electrical disease (palpitations, presyncope, syncope, resuscitated SCD) [6]. For the detection of electrical disease, **twelve-leads ECG** is a sensitive diagnostic tool, able to identify early signs of the disease, such as 1^st^ degree AVB or various kinds of inter/intraventricular conduction delays [28]. However, it may lack sensitivity for the detection of paroxysmal brady or tachyarrhythmias. To overcome that limitation, **24/48-hour Holter monitoring or long-term event monitors** are of particular interest to identify second or third degree AVBs, supraventricular tachyarrhythmias, PVCs, NSVT and even sustained VA [23,30]. Probably, implantable loop recorders would give the best sensitivity in detecting paroxysmal arrhythmias, but ad-hoc studies are still lacking so far. By converse, the role of **invasive electrophysiologic evaluation** for both conduction system disease characterization and for tachyarrhythmias risk stratification is still under debate but probably non-necessary or even misleading [26].

As for the mechanical phenotype, assessment of left ventricular function to determine left ventricular dimension and function is recommended. **Two-dimensional echocardiography** is regarded as the standard method to clinically study DCM [31]. In addition, echocardiogram also allows for the evaluation of diastolic function and chamber volumes, being also able to exclude secondary causes of DCM (like infarction scar, significant valvulopathies, or abnormal loading conditions) and to monitor cardiomyopathy evolution to plan appropriate timing for HTx. However, as already discussed, LV size and function may be near-normal, especially in the early phase of the disease [22]. Thus, more sophisticated techniques like speckle tracking have recently been proposed to identify early markers or the disease, such as longitudinal LV systolic function impairment or increased mechanical dispersion at segment-by-segment longitudinal strain analysis [32].

Among second-level imaging, **cardiac magnetic resonance** (CMR) plays an important role in tissue characterization (or ‘*in vivo* histology’) of patients with LMNA-related DCM [8,33,34], allowing the detection of gross myocardial fibrosis by late gadolinium enhancement (LGE) technique [8,34]. In particular, mid-myocardial enhancement in a non-coronary distribution, typically linear and less than 50% of the area of each segment, has been demonstated in these patients, predominantly in the LV basal and mid-ventricular septum, with a patchy or mid wall pattern [34]. However, since gross replacement fibrosis targeted by LGE is more commonly detected in carriers already expressing the typical cardiac phenotype, new CMR technologies are nowadays called to identify interstitial myocardial fibrosis, which may represent a subclinical marker preceding the overt structural, functional, and electrical abnormalities [8]. This is the case of T1 mapping technique, which has shown to be able to detect interstitial fibrosis by comparing extracellular/extravascular volume fraction of the myocardium pre- and post-contrast, with significant preliminary results in familial DCMs [35]. Consistently, **electroanatomical mapping** in LMNA patients affected by VAs showed the presence of low voltage areas, as electrical expression of fibrosis consistent with the origin of clinically manifest VTs: in 82% of patients the scar was found in the basal left ventricle, particularly in the septum, followed by basal inferior wall and subaortic mitral continuity [10].

Invasive **endomyocardial biopsy** with standard histological analysis, however, may show cardiomyocyte hypertrophy and interstitial fibrosis with focal replacement scarring, not differently from other DCMs [14]. In addition, in some LMNA mutations fibrosis is particularly significant [7]. Fibro-fatty degeneration and atrophy have been observed in the atrioventricular node of diseased patients, but this finding comes from explanted hearts and cardiac conduction system cannot be part of diagnostic biopsy sampling [27]. Only conventional electron microscopy and immuno-electron microscopy allows for identifications of ultrastructural nuclear membrane changes and loss of protein expression typical of LMNA-related DCM: among them, incomplete nuclear membrane rupture, bullae, and disorganization of the nuclear pores [14,36]. However, endomyocardial biopsy is an invasive technique, and nowadays far from a true clinical usefulness.

Non-significant alterations have been reported in **blood exams** too. Both T and I troponin may be used as biomarkers of acute or ongoing myocardial damage, as generally shown in non-ischemic DCMs and HF patients, but lack sensitivity and specificity [37,38]. By converse, the natriuretic peptide NT-proBNP, released in response to wall stress from volume overload, has shown to correlate with LV and/or right ventricular dysfunction in patients with LMNA mutations [39]. However, NT-proBNP is often elevated in many other DCMs or conditions leading to HF; thus, even if it seems to have some role in risk stratification in LMNA-related cardiomyopathy, its diagnostic usefulness has not been proved so far. Finally, measurement of serum CK concentration may be useful to evaluate the possible coexistence of subclinical skeletal myopathy, in order to schedule a neuromuscular specialistic evaluation as indicated [11].

Last but not least, **genetic testing** is obviously the key diagnostic tool to determine the disease etiology. The diagnosis of laminopathy is established in a proband by the identification of a heterozygous pathogenic variant in the LMNA gene. Molecular genetic testing approaches can include the use of a multi-gene panel or a more comprehensive genomic testing, like whole-exome sequencing and whole-genome next generation sequencing [40]. Once a LMNA pathogenic variant has been identified in a proband, targeted molecular genetic testing can be offered to relatives in order to facilitate the pre-clinical identification of at risk subjects [6]. Current limits of genetic analysis are linked to difficulties in variant interpretation associated with the identification of variants of unknown significance [41]. In these cases, careful evaluation of variant segregation in families may help to assign a pathogenetic role.

## Risk stratification

Due to the rarity of the disease and to its heterogeneous phenotype, large prospective longitudinal studies on LMNA-related cardiomyopathy are not yet available so far. Most of the evidence about the natural history of the disease comes from single-center experiences or multicenter retrospective registries. The prognosis of LMNA patients is worse compared to other familial DCMs: in fact, by the age of 45 years, carriers of LMNA mutations presented cardiovascular death, HTx, or at least one major event (hospitalization for HF deterioration, VA or thromboembolic event) in 69% vs. 25% in familial DCM patients without LMNA mutations [4]. Even though no clear genotype-phenotype correlations have been established to date, in a previous paper we observed that LMNA patients with adult onset mainly showed cardiac disease associated with frameshift **mutations**, suggesting that late onset phenotypes with cardiac involvement may arise through loss of function of lamin A/C protein secondary to haploinsufficiency, while dominant negative or toxic gain of function mechanisms may explain the severity of early phenotypes [42]. Moreover, genetic analysis has shown to be helpful in SCD risk stratification alone: in fact, a few recent studies focusing on pathogenic variant type suggest a correlation between splice site variants and increased risk for SCD and more in general between non-missense variants (insertion/deletion, truncating or splice site) and risk for malignant VA [21,23,30]. Finally, some specific mutations have been implicated in a worse prognosis, like the point mutation Asn195Lys, which is characterized by a higher incidence of both SCD and HTx risk, and the point mutation Arg225X, with more frequent conduction system disease [43]. Also **gender** seems to play a role in risk stratification [23,30]: in a multicentre cohort of 269 LMNA mutation carriers, in particular, authors showed that male individuals have a worse prognosis due to higher prevalence of malignant VA and end-stage HF [16].

Considering **mechanical** endpoints, since manifest structural heart disease seems to appear as a late phenomenon, the usefulness of imaging-driven morphological and functional predictors in clinical risk stratification results pretty low. Nevertheless, early signs of mechanical dysfunction have recently been identified as possible risk predictors, as shown above. However, they often require sophisticated technologies like echocardiographic softwares for speckle tracking and strain analysis or CMR, which are not easily available widespread, thus limiting their clinical usefulness [8,32,34,44]. Clinical equivalents of mechanical dysfunction, like New York Heart Association (NYHA) HF functional class have shown some prediction capability in a study [21]; however, NYHA class seems a pretty late endpoint (NYHA classes III and IV only resulted predictive), non-completely reproducible and mainly not confirmed in later studies [22,30]. Similarly, preliminary results about the role of natriuretic peptide NT-proBNP in predicting biventricular dysfunction in patients carrying LMNA mutations are still to be confirmed before considering it as a reliable prognostic biomarker [39].

Given the late manifestation of structural endpoints, even SCD prediction is a trouble, since the classical risk stratification strategy based on LVEF and NYHA class to indicate ICD implant in primary prevention cannot be applied to LMNA-related cardiomyopathy. In fact, as shown above, up to 1/3 of LMNA mutation carriers who manifested sustained VA had preserved ventricular function (LVEF > 50%), and 56% did not meet conventional criteria for ICD implantation because they had LVEF > 35% at the time of first sustained VA [23]. By converse, deeply to anatomical substrate, **LGE** at CMR resulted a significant predictor of multiple events. First, LGE has been associated with a significantly higher incidence of not only LV dilatation and/or dysfunction [8], but also diastolic dysfunction [45]. Second, LGE positively correlates with conduction system disease, in particular 1^st^ degree AVB with great sensitivity: in fact, the occurrence of conduction abnormalities correlated significantly with presence of LGE in the LV septum, even with normal ECG recording [33]. Finally, it has been reported that mid-wall myocardial fibrosis, as assessed by LGE, predicted both VT and SCD in DCM patients [34,46].

Among pure electrical predictors, **minor conduction defects**, such as first degree AVB, a widened QRS complex **and supraventricular tachyarrhythmias like** atrial fibrillation, correlated with the presence of septal wall fibrosis and local motion abnormalities in some studies, thus contributing at least indirectly in prognosis prediction [26,47]. In EDMD patients with no structural heart disease, simple ECG parameters indicating heterogeneity of atrial activation (maximum P-wave duration, P-wave dispersion) and ventricular repolarization (QTc and JTc dispersion), have been identified as possible predictors of atrial tachyarrhythmias and malignant VA, respectively [48,49]. More significantly, **minor VA** like NSVT detected by 24/48 h Holter monitoring seem to play as a reliable prognostic indicator especially for the prediction of malignant VA [22,30]. By converse, even though electrophysiological study has been proposed before the implantation of a pacemaker or for screening purposes at the age of 35, it is still doubtful whether it can stratify risk for SCD, so there are currently no indications for its use in LMNA patients [22,26,50].

These data suggest that an **integrated model** may be appropriate for risk stratification in patients with LMNA-related cardiomyopathy: in fact, results from the largest retrospective studies in this field suggest to adopt a multiparametric criterion to predict prognosis. This is the case of a multicentre registry of 269 LMNA mutation carriers, in which malignant VA occurred only in patients with at least two of the following risk factors: NSVT during ambulatory Holter ECG monitoring, LVEF < 45% at first clinical evaluation, male gender and non-missense mutations [30]. Based on that study, the most recent international guidelines on VA and SCD suggest ICD implantation in LMNA mutation carriers with DCM and clinical risk factors defined as above (recommendation IIa, level of evidence B) [51]. Thus, differently from other DCMs, ICD implantation should be considered even before the ejection fraction falls below 35%, in the presence of known risk of arrhythmia [6]. More recently, in a multicentre registry of 122 LMNA mutation carriers, followed for a median of 7 years from first clinical contact, male sex, nonmissense mutations, and LV dysfunction (defined with the very early cutoff of 50% instead of 45%) at index evaluation were associated with development of VA, whereas LV dysfunction alone was associated with end-stage HF or death [23]. Significantly, the mode of presentation (with isolated or combination of electrical and mechanical clinical features), did not predict sustained VA or end-stage HF or death. Of note, however, possible extracardiac predictors like associated neuromuscular phenotype have never been considered so far in LMNA-cardiomyopathy risk stratification.

## Follow-up and therapy

Because of the complexity of LMNA-related heart disease in both symptomatic and asymptomatic individuals, referral to centers with special expertise in cardiovascular genetic medicine and electrophysiology should be considered. Individuals with a LMNA pathogenic variant who are found to have any ECG abnormality should undergo cardiovascular evaluation for disease progression at least annually [52]. This should include, minimally, a 12-leads ECG, rhythm monitoring by 24/48 hour Holter ECG and measurement of biventricular size and function through echocardiogram. If available, CMR and device interrogation data would be helpful in adding precious diagnostic and prognostic information. A complete summary of diagnostic tools and suggested follow-up frequencies are reported in Table 1.

**Table 1.** Diagnostic tools in cardiac laminopathies. The table shows the main diagnostic tools in cardiac laminopathies, with most common findings, the main detectable events together with their predictors, the subsequent indication according to the main studies published so far and the suggested follow-up calendar. *Abbreviations: AF/Flu = atrial fibrillation/flutter; AVB = atrioventricular block; CMRI = cardiac magnetic resonance; DCM = dilated cardiomyopathy;EMB = endomyocardial biopsy; IVCD = interventricular conduction defects; LA = left atrium; LBBB = left bundle branch block; LGE = late gadolinium enhancement; LV = left ventricle; LVEF = left ventricular ejection fraction; NSVT = non-sustained ventricular tachycardia; PVC = premature ventricular complex; RV = right ventricle; SSS = sick sinus syndrome; VT/VF = ventricular tachycardia/fibrillation.*

	Findings	Predictors of events	Events	Indication	Calendar minimal frequency
12-leads ECG	Any degree AVB, SSS, LBBB/IVCD	First degree AVB	High degree AVBs	Strong	Twice a year
24/48 h Holter monitoring	SSS, any degree AVB, PVC, NSVT, VT/VF, SVA	NSVT	VT/VF	Strong	Twice a year
Device interrogation (when applicable)	NSVT, VT/VF, SVA	NSVT	VT/VF	Strong	Twice a year
TTE	LV dilatation/dysfunction, RV and LA morphology and function	LVEF < 45–50% Strain	DCM	Strong	Twice a year
Genetic test	Aetiologic definition	Non-missense mutations	VT/VF	Strong	Once in lifetime
CMR	Biventricular morphology and function, LGE	LGE	AVB, VT/VF, DCM/HF	Medium	Once in lifetime
EMB	Fibrosis	Fibrosis	AVB, VT/VF, DCM/HF	Low	No
Electroanatomical mapping	Low-voltage areas	/	VT/VF	Low	No
Electrophysiological study	VT/VF inducibility, conduction intervals	/	AVB, VT/VF	Low	No

Whether conventional medical therapy (ACE inhibitors, beta blockers) is able to improve or prevent **DCM or HF** in LMNA-related heart disease has not been formally tested and is therefore unknown. However, in patients with symptomatic DCM, optimal medical treatment for HF is probably indicated, not differently from other forms of DCMs [53,54]. It is worth noting, however, that given the intrinsic frequency of bradyarrhythmias observed in LMNA-related DCM, all of the drugs with a negative chronothropic effect (like beta blockers or non-didyidropyride calcium channel blockers) should be used carefully, or possibly avoided unless an electronic pacemaker is in place [6]. Symptomatic drugs like diuretics, and other conventional therapy for HF should be used according to updated guidelines [54]. Similarly, HTx or other advanced therapies should be considered with progressive DCM, advancing HF, and otherwise refractory disease [55].

Evidence is lacking also for **arrhythmias** management, in particular about optimal antiarrhythmic drug therapy. Atrial fibrillation unresponsive to cardioversion is treated with anticoagulants and agents for ventricular rate control [56]. Other symptomatic supraventricular arrhythmias are treated basically with pharmacologic agents too, since no studies about invasive ablation procedures in supraventricular tachycardias have been reported so far [57]. With progressive deterioration in left ventricular function (LVEF < 30%) while still in sinus rhythm, some experts recommend full anticoagulation to prevent the development of left ventricular mural thrombosis and systemic embolic events including stroke [6,23]. It is worth noting, in general, that a significant occurrence of thromboembolic disease is reported in patients with LMNA mutations, so that probably indication to anticoagulation should be taken into account in the presence of risk factors for thrombosis [58]. Symptomatic bradyarrhythmias or even asymptomatic high-degree AVBs should be treated with an implantable electronic pacemaker, as indicated [59]. However, every time a device is indicated for pacing, the implant of an ICD rather than an electronic pacemaker is recommended, given the high incidence of malignant VAs intrinsically due to LMNA-related disease [47,51]. It has been shown, in fact, that 42% of the patients undergone ICD implantation at the time of appearance of the conduction defect, had a VT successfully treated during the follow up [60]. For the same reasons, it seems appropriate to implant a CRT-D every time a CRT-P is indicated for patients with structural heart disease. Symptomatic VA, VT, VF and resuscitated SCD are treated with an ICD and drug therapy as needed, since implant in secondary prevention is undoubtful [6,51]. As for primary prevention, early ICD implantation in LMNA mutation carriers with minimally impaired LVEF and no indications to cardiac pacing may be a reasonable treatment, given the rapid deterioration in LV function and the high arrhythmic risk [23,61]. At the moment, risk factors suggesting early ICD implantation have been proposed to be non-missense mutations, male gender, LVEF < 45% and detection of NSVT [30].

Catheter ablation of VT associated with LMNA cardiomyopathy has been recently shown to be associated with a poor outcome, including high rate of arrhythmia recurrence, progression to end-stage HF, and high mortality (26%) [10]. Moreover, multiple VTs were inducible per patient and noninducibility of any VT was achieved in 1/4 of patients only, so that multiple procedures were required (median 2/patient). Notably, acute procedural insuccess was attributed to intramural septal substrate in 72% of the patients [10]. Based on these results, VT ablation is probably not indicated, with the possible exception of controlling VA in patients suffering from electrical storms and multiple appropriate shocks after ICD implantation, despite optimal medical therapy.

**Figure 2.**
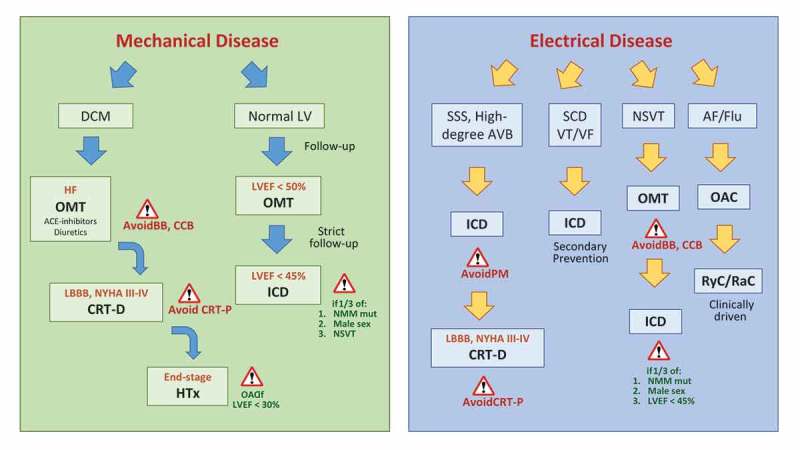
Mechanical and electrical disease management in cardiac laminopathies. The figure shows the best clinical management for patients presenting with mechanical (green panel, on the left) or electrical disease (blue panel, on the right), according to the main studies published so far. *Abbreviations: AF/Flu = atrial fibrillation/flutter; AVB = atrioventricular block; BB = betablockers; CCB = calcium-channel blockers; CRT = cardiac resynchronization therapy with (CRT-D) or without (CRT-P) defibrillator function; DCM = dilated cardiomyopathy; HF = heart failure; HTx = heart transplantation; ICD = implantable cardioverter defibrillator; LBBB = left bundle branch block; LV = left ventricle; LVEF = left ventricular ejection fraction; NMM = non-missense mutation; NSVT = non-sustained ventricular tachycardia; OAC = oral anticoagulants; OMT = optimal medical therapy; PM = pacemaker; RyC/RaC = rhythm/rate control; SSS = sick sinus syndrome; VT/VF = ventricular tachycardia/fibrillation.*

An integrated approach to the electrical and mechanical management of LMNA-related cardiomyopathy is reported in Figure 2.

## Future therapeutic perspectives

No gene or biological targeted therapy is nowadays available for the early treatment of LMNA-associated cardiac disease. However, great expectancy arises from basic research on laminopathies. In fact, understanding how lamins control and alter gene expression and signaling pathways holds great potential for therapeutic application also in LMNA cardiomyopathies [62]. In particular, modulation of mTOR signaling currently provides the most promising way forward for the treatment of LMNA cardiomyopathy in humans, with preliminary data showing improved cardiac function in patients with structural heart disease [62,63]. Also animal models are being used to study molecular pathways affected by LMNA mutations, in particular those regulating cell proliferation, growth, differentiation, survival, migration, and apoptosis [64,65]. For example, since inappropriate phosphorylation of ERK1/2 and subsequent gap junction dysfunction may contribute to arrhythmogenesis, tageted therapy on junction protein connexin43 may find application in cardiac laminopathies, as already shown [66]. Based on available preliminary results, it is possible to hypothesize that, in the next years, new therapeutic options will result from the strong interplay between basic and clinical research.

## Conclusion

Cardiac laminopathy still represents a complex disease, requiring special considerations and a global evaluation of both the electrical and mechanical status of the heart. Further research will be necessary to describe long term natural history by larger multicenter long follow-up studies, to find new predictors and earlier signs of the disease and to work out novel drugs and therapeutic strategies. A particular effort should then be made in stratifying risk in asymptomatic mutation carriers, since relatives very often seek medical counselling after a mutation is discovered in a proband; actually, although currently the same disease predictors apply to both probands and relatives, it seems that asymptomatic mutation carriers often present no pathologic phenotype even at long-term follow-up. Finally, since laminopathies are complex diseases potentially involving multiple systems, multispecialistic evaluation is required, also in patients apparently presenting with heart involvement only. Among the others, interesting results may come from detailed neuromuscular characterization of patients suffering from LMNA-related cardiomyopathy, in order to find out new possible relationships and better stratify their prognosis.
